# Inhibition of Glioma Cell Lysosome Exocytosis Inhibits Glioma Invasion

**DOI:** 10.1371/journal.pone.0045910

**Published:** 2012-09-28

**Authors:** Yu Liu, Yijiang Zhou, Keqing Zhu

**Affiliations:** Department of Pathology, Department of Neurobiology, Zhejiang University School of Medicine, Hangzhou, China; Complutense University, Spain

## Abstract

Cancer cells invade by secreting enzymes that degrade the extracellular matrix and these are sequestered in lysosomal vesicles. In this study, the effects of the selective lysosome lysing drug GPN and the lysosome exocytosis inhibitor vacuolin-1 on lysosome exocytosis were studied to determine their effect on glioma cell migration and invasion. Both GPN and vacuolin-1 evidently inhibited migration and invasion in transwell experiments and scratch experiments. There are more lysosomes located on the cell membrane of glioma cells than of astrocytes. GPN decreased the lysosome number on the cell membrane. We found that rab27A was expressed in glioma cells, and colocalized with cathepsin D in lysosome. RNAi-Rab27A inhibited lysosome cathepsin D exocytosis and glioma cell invasion in an *in vitro* assay. Inhibition of cathepsin D inhibited glioma cell migration. The data suggest that the inhibition of lysosome exocytosis from glioma cells plays an important modulatory role in their migration and invasion.

## Introduction

The activation and release of proteases from cancer cells induces invasive, migratory behavior in vitro and metastasis in vivo [1]. Because these proteases are sequestered in lysosomes, lysosomes may be key mediators of protease release in cancer cell invasion [2]. Lysosomes play a pivotal role in the degradation of extracellular matrix (ECM) proteins, cell invasion, and cell migration into the ECM because several of the proteases that contribute to ECM degradation are directly or indirectly associated with lysosome exocytosis [3], [4]. The lysosomal cathepsins are a major class of matrix-degrading enzymes involved in tumor invasion. For instance, cathepsin D, which is sequestered in lysosomes, exhibits proteolytic activity when activated by the acidic lysosomal environment. Clinically, the level, activity and localization of cathepsins is of diagnostic and prognostic value. For example, Cathepsin D is a potential serum marker for poor prognosis in glioma patients [5], [6]. Inhibition of the exocytosis of proteases from cancer cell lysosomes could lead to the development of an efficacious therapy for cancer.

Gliomas are the most frequently diagnosed primary brain malignancy. These tumors have a tendency to invade diffusely into surrounding healthy brain tissue, thereby precluding successful surgical removal. In this study, we selected the glioma cell lines as the model and investigated the potential roles of selective lysosome lysis and inhibition of lysosome exocytosis in this process by modulating glioma cell migration and invasion [7].

The small G proteins of the Rab family regulate discrete steps in vesicular transport pathways. Recent studies showed that one member of this family, Rab27A, regulates the transport of lysosome-related organelles [8], [9]. Secretory lysosomes have the capacity for regulated exocytosis [10]. Downregulation of Rab27a, required for the trafficking of secretory lysosomes to the plasma membrane, blocked lysosome exocytosis. To avoid the possible non-selective effects of GPN and vacuolin-1 on the inhibition of lysosome exocytosis, we assessed the involvement of Rab27A in lysosome-related glioma cell invasion.

**Figure 1:**
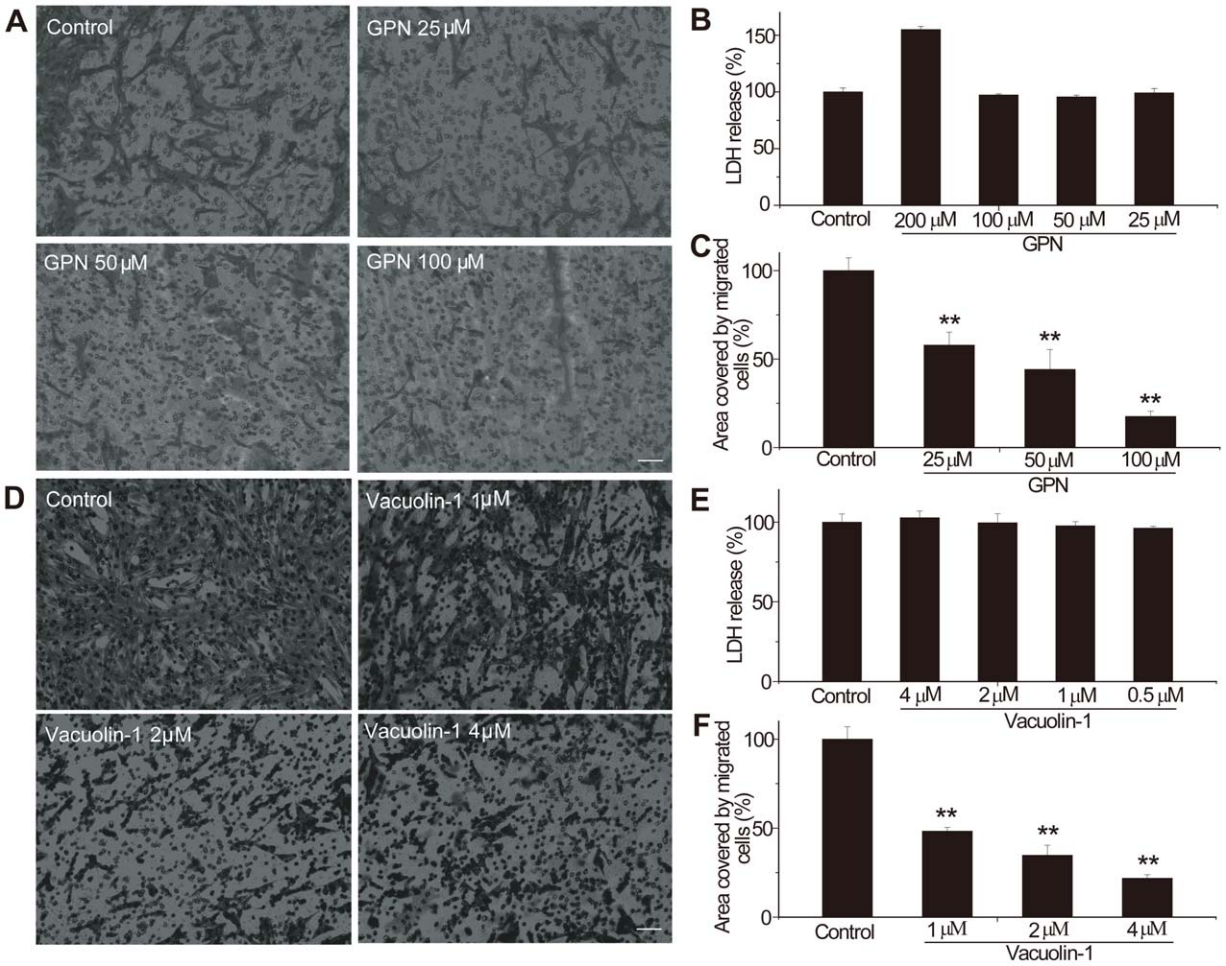
GPN (A–C) and vacuolin-1 (D–F) inhibit C6 glioma cell invasion and migration in transwell assay. A. Images of C6 glioma cells on the undersurface of a filter which were either untreated (control) or treated with different doses of GPN. The images show that the number of cells passing through the filter with ECM gel decreased with GPN treatment. B. LDH assays showing 25–100 µM GPN was not toxic to the C6 cell line, while 200 µM GPN did have a toxic tendency. Bars represent the average ± S.E., n≥3. Magnification, ×20. C. The area covered by migrated cells in control, untreated samples was set at 100%, and the change for samples treated with GPN was 57.99±7.32% for 25 µM GPN, 44.20±11.18% for 50 µM GPN, 17.54±3.03% for 100 µM GPN, and 9.55±3.77% for 200 µM GPN (p<0.01). Bars represent the average ± S.E., n≥3. Magnification, ×20. D. Images of cells on the undersurface of a filter, either untreated (control) or treated with different doses of vacuolin-1. As the vacuolin-1 dose increased, the number of cells on the undersurface of the filter decreased. E. LDH assays showing 0.5–4 µM vacuolin-1 was not toxic to the C6 cell line. Bars represent the average ± S.E., n≥3. Magnification, ×20. F. Quantification of transwell assays. The overall change in the surface area with untreated samples was 100%, and the change in surface area for samples treated with vacuolin-1 was 67.40±2.96% at 1 µM, 39.18±3.54% at 2 µM, and 43.20±3.80% at 4 µM (p<0.01).

Our research aim was to determine if inhibition of lysosome exocytosis from glioma cells inhibits their migration and invasion. Here, we showed that the inhibition of lysosome exocytosis by chemicals or RNAi inhibited glioma cell migration and invasion. Functionally, we demonstrated that RNAi-Rab27A inhibited exocytosis of the lysosome enzyme cathepsin D and inhibition of cathepsin D enzyme activity inhibited glioma cell migration. Furthermore, Rab27A and cathepsin D colocaolized in glioma cell lysosomes. More lysosomes appeared on the glioma cell surface than on astrocytes, and GPN decreased the cell surface lysosome appearance. The results suggested that inhibition of lysosome exocytosis might be a rational approach to the clinical treatment of glioma.

**Figure 2:**
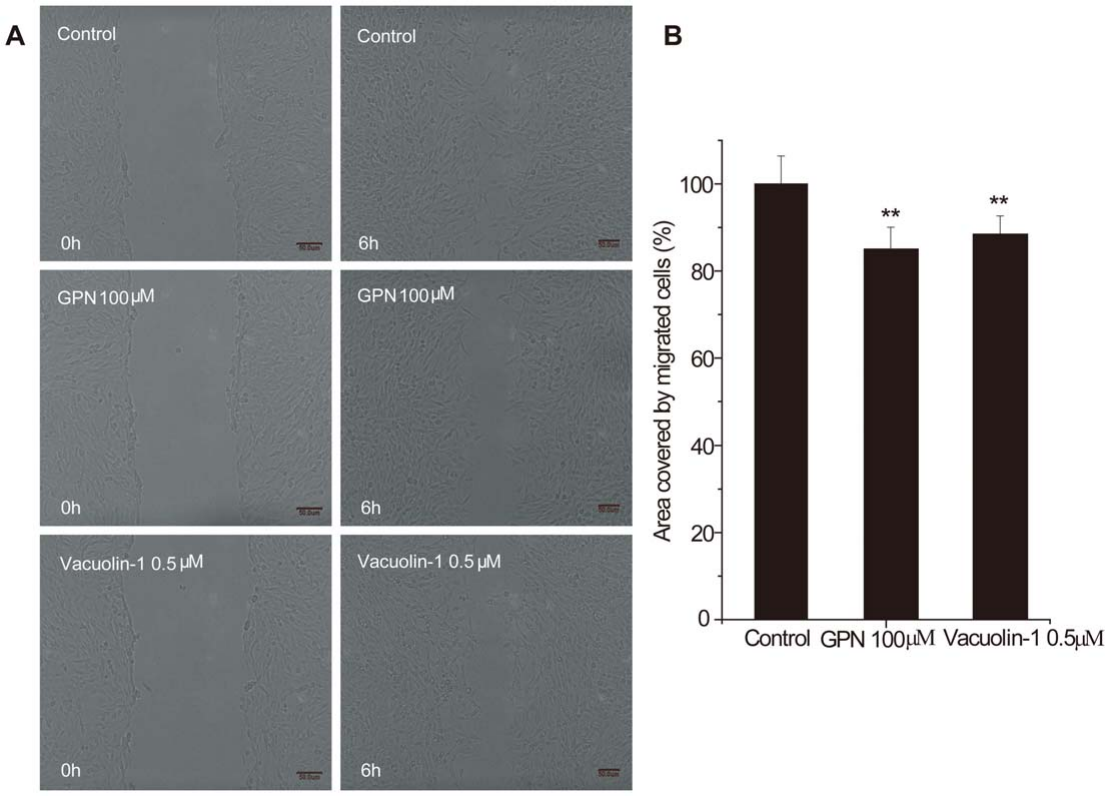
GPN and vacuolin-1 inhibit C6 glioma cell migration in scratch assay. A. Images taken at the initiation of the experiment (t = 0) and after 6 h (t = 6). Movement of cells into the scarred region resulted in a decrease in the surface area of the scar. B. Statistical summary. The surface area of the scar for samples treated with 100 µM GPN was 85.04±0.05%, and with 0.5 µM vacuolin-1 was 88.45±4.22% (p<0.01). Bars represent the average ± S.D.; results in B are from a representative experiment (n = 3). Magnification, ×20.

## Methods

### Cell Culture

The C6 and U251 glioma cell lines (from the American Type Culture Collection) were maintained in DMEM (Invitrogen Corp.) supplemented with 10% fetal bovine serum, and 100 U/ml penicillin (Invitrogen). All cell lines were kept in a humidified atmosphere of 5% CO_2_ at 37°C. Glycyl-L-phenylalanine-ß- naphthylamide (GPN) and vacuolin were from Sigma (St. Louis, MO).

**Figure 3:**
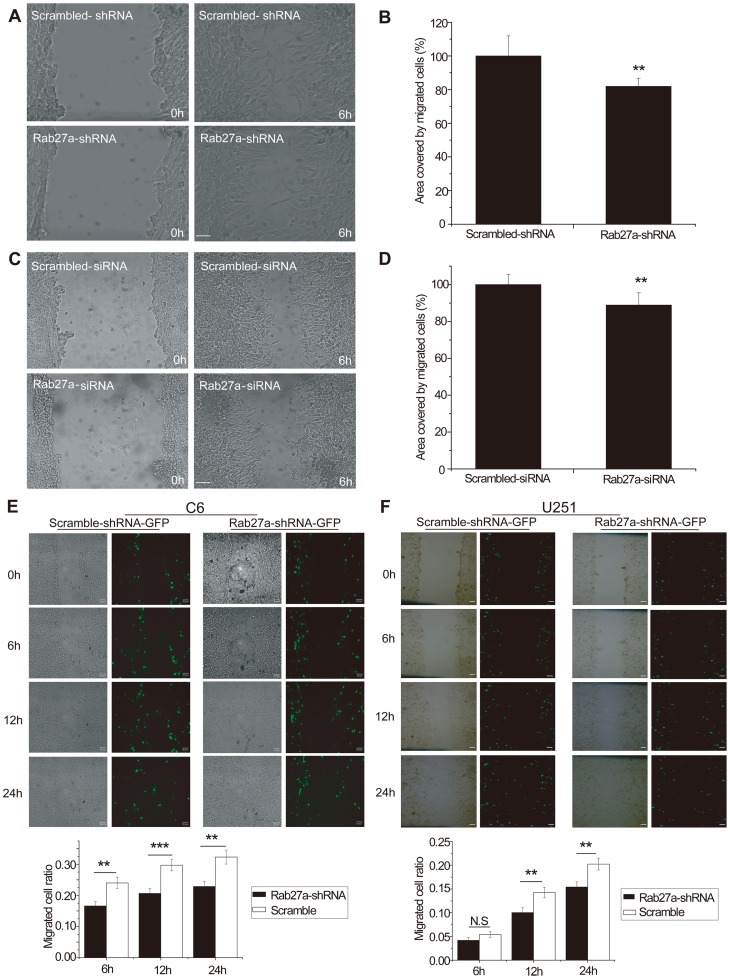
RNAi-Rab27a inhibited glioma migration in scratch assay. Both Rab27a shRNA (A) and Rab27a siRNA (C) decreased C6 cell migration 6 h after scratching. The change in the surface area of the scar for samples treated with shRNA was 70.36±3.06% (B), and with siRNA was 88.88±5.95% (D) (p<0.01). E. Rab27a shRNA green fluorescent C6 cells migrated more slowly than the scrambled controls at all time points (6, 12, and 24 h; p<0.01). F. Rab27a shRNA green fluorescent human U251 cells migrated more slowly than the scrambled controls at all time points (6, 12, and 24 h; p<0.01).

**Figure 4:**
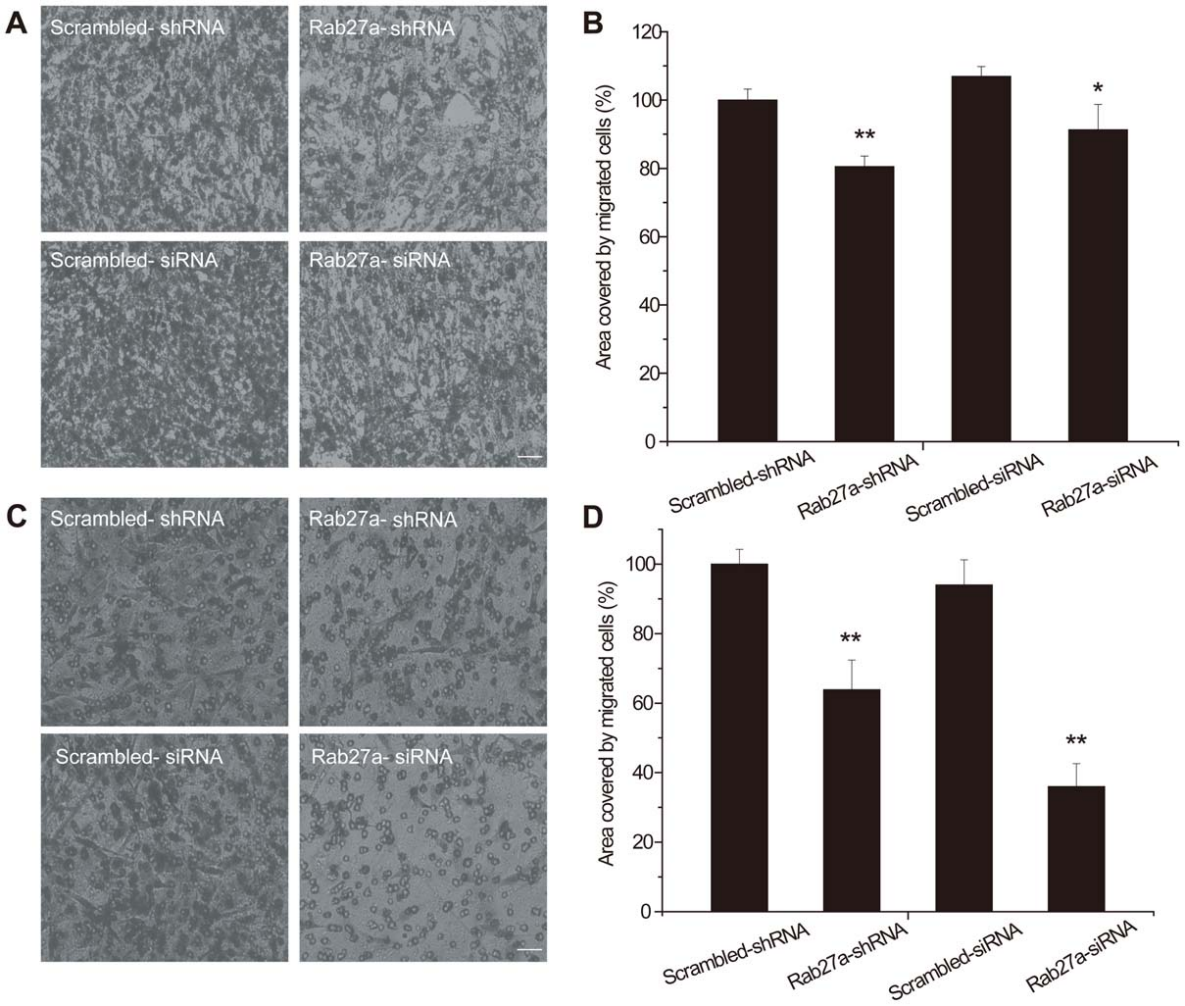
RNAi Rab27a inhibited C6 glioma cell migration and invasion in transwell experiments (with or without ECM gel). A. Images of cells on the undersurface of a filter without ECM gel, either untreated (control) or treated with Rab27a shRNA or Rab27a siRNA, showing that the number of cells decreased. B. Quantification of the transwell assays. The surface area for samples treated with Rab27a shRNA was 80.53±3.06%, and with Rab27A siRNA was 85.40±6.94% (p<0.01). C. Images of cells on the undersurface of a filter with ECM gel, either untreated (control) or treated with Rab27a shRNA or Rab27a siRNA, showing that the number of cells decreased. D. Quantification of the transwell assays. The change in the surface area for samples treated with Rab27a shRNA was 63.89±8.49%, and with Rab27a siRNA was 36.04±6.59% (p<0.01). Bars represent the average ± S.E., n≥3. Magnification, ×20.

### Scratch assay

The scratch motility assay was used to measure two-dimensional movement [11]. C6 or U251 glioma cells were grown to confluence in 6-well plates. A scratch was then made on the monolayer using a sterile 200-µl pipette tip. Medium containing serum and inhibitors was added and cells were incubated at 37°C. For RNAi experiments, the cells were treated with siRNA or shRNA as described and the monolayers were scratched 2–3 days after transfection. For the cathepsin D inhibitor pepstatin A experiment, cells were pre-treated with 10 μM pepstatin A or 0.05% DMSO for 3 h. Cells were photographed at different time points and the scratch area was measured using ImageJ. At the initiation of the experiment (t = 0) a digital image of the scar was taken at a magnification of ×10 (Axon Imaging, Foster City, CA). After 6 h (t = 6) the same region of the scar was imaged again. The ImageJ software allowed us to quantify the two-dimensional movement of the cells by measuring the surface area of the scar at t = 0 and comparing it with the surface area at t = 6. Experiments were repeated at least three times. Measurements were made in triplicate.

**Figure 5:**
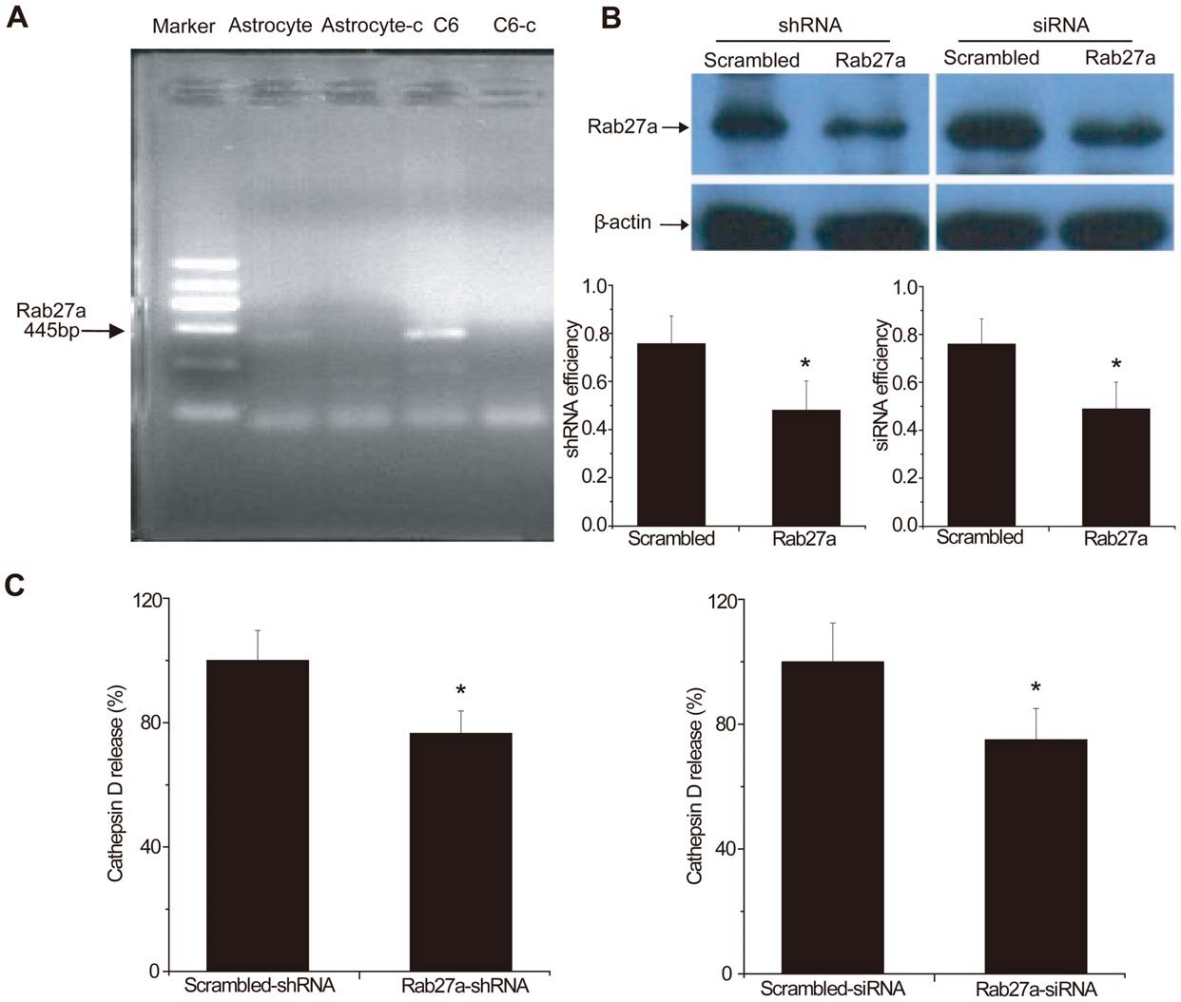
RNAi Rab27a effect on cathepsin D release from C6 glioma cells. A. Images of RT-PCR showing Rab27a expressed in astrocytes and C6 glioma cells. There was a single, distinct PCR product at approximately 445 bp when either astrocyte cDNA or C6 cDNA was used. B. Western blots showing decreased protein expression by both Rab27a shRNA and Rab27a siRNA treatment. Quantification showing that the downregulation was 30–40%. C. The cathepsin D release ratio for Rab27a shRNA group was 76.55±7.22%, and for Rab27a siRNA group was 75.00±10.01%, relative to the control group. Both the Rab27a shRNA and siRNA groups decreased cathepsin D release by ∼25%.

**Figure 6:**
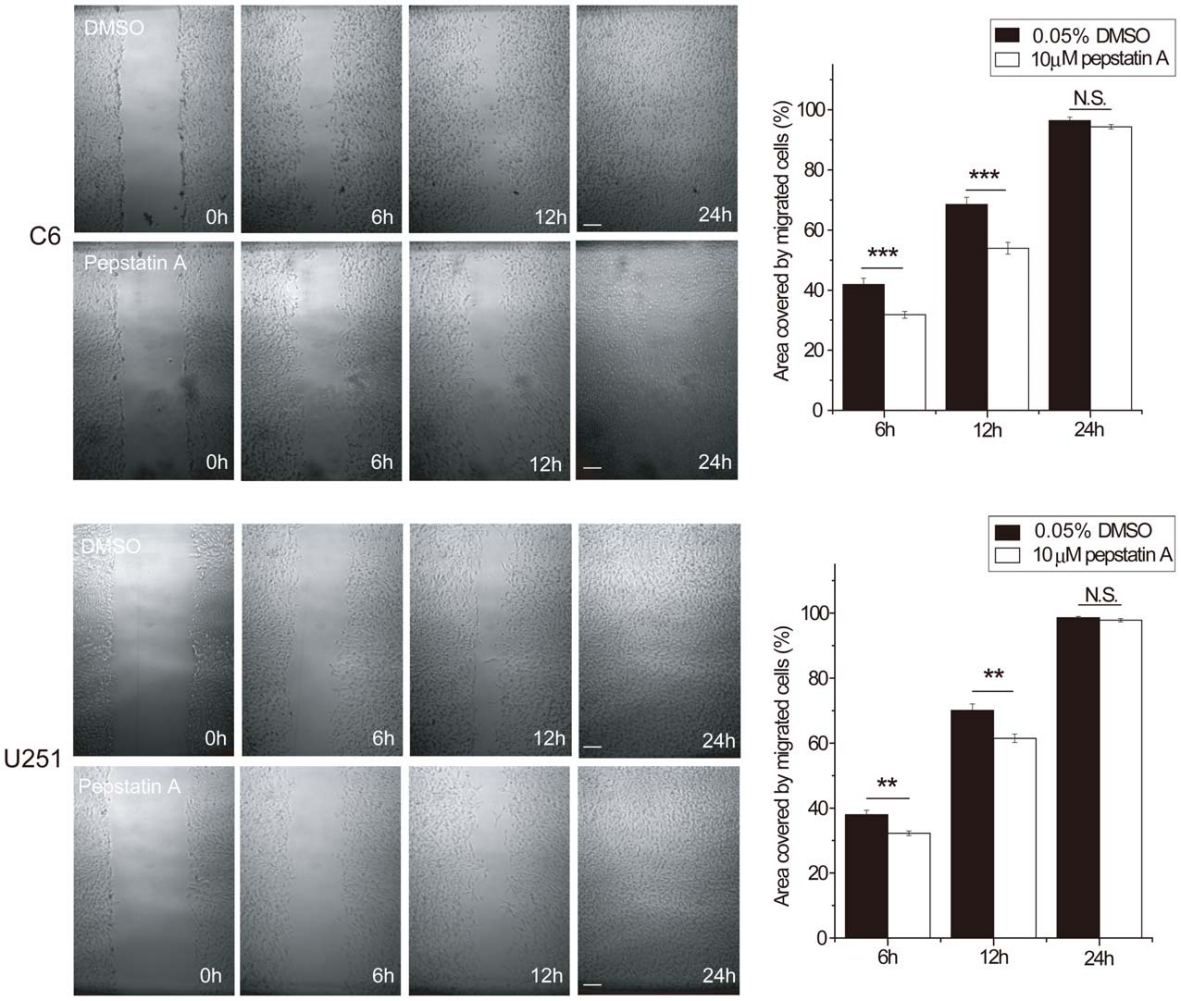
The cathepsin D inhibitor pepstatin A inhibited glioma cell migration in scratch assay. Both C6 and U251 cells migrated more slowly than the controls at the time points (6, 12 h; p<0.01).

### Transwell assay

Cell migration was assayed in triplicate using a 24-well transwell setup (Neuroprobe) using polycarbonate Nucleopore filters with an 8-μm pore size. The underside of the membrane was precoated with (invasion model) or without (migration model) ECM gel (Sigma) in DMEM for 16 h at 4°C. Cells were centrifuged, resuspended in serum-free medium and 15,000 cells were seeded in the upper chamber of each well. The lower chambers contained serum-free medium. The cells were rinsed with PBS, fixed, and stained with an ethanol-based crystal violet solution after the inhibitors (GPN or vacuolin-1) were added for 16–24 h. Non-migrated cells on the upper side of the membrane were removed by scraping, while migrated cells attached to the underside were fixed for 10 min in methanol and stained with crystal violet (Sigma). Cells were examined under a microscope and all cells in a specified area in the middle of the membrane were counted. Experiments were repeated at least three times.

**Figure 7:**
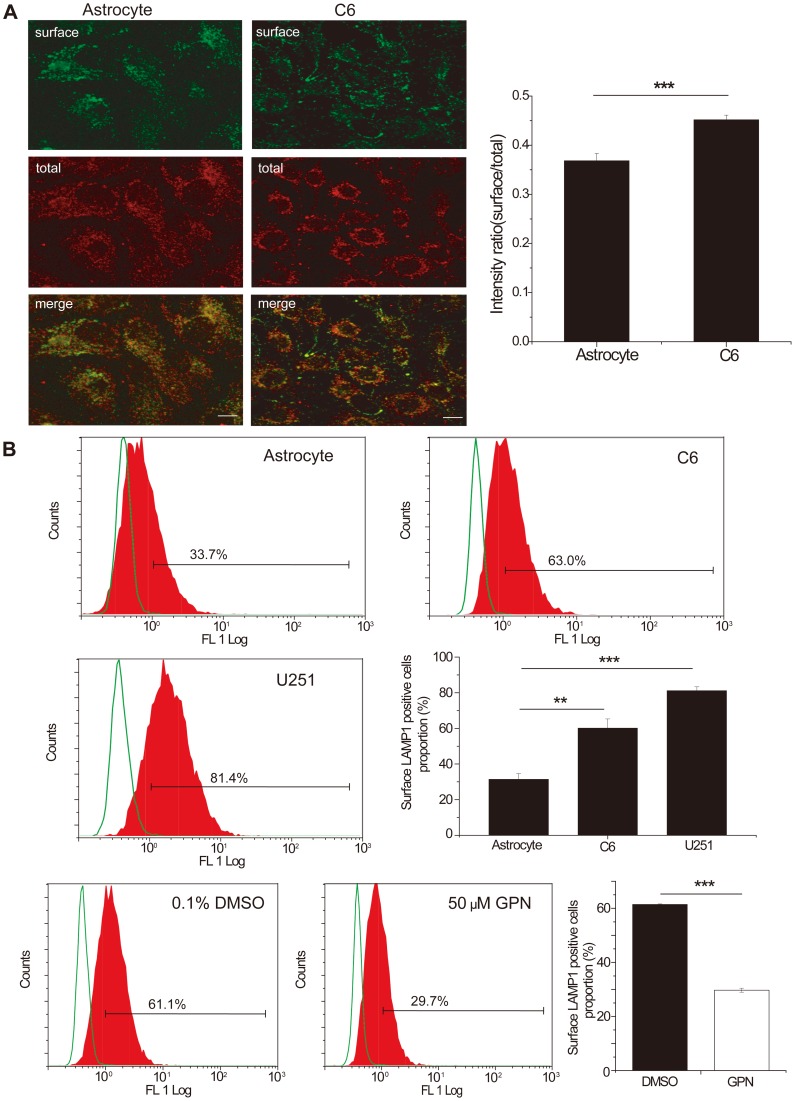
LAMP1 surface staining showing more lysosomes near the glioma cell surface than in astrocytes. GPN treatment decreased glioma cell surface lysosome number. A. Upper: representative confocal images depicting the astrocyte surface Lamp1 (green) and the total Lamp1 (red). Lower: quantification of the intensity ratio (surface/total). Error bars represent the s.e.m. of 42 images from two independent experiments; ***denotes P<0.001 of C6 compared with astrocytes. (Student's t-test). B. The FACS results of LAMP1 surface staining showing more lysosomes on the surface in both C6 and U251 glioma cells than in astrocytes. GPN treatment decreased LAMP1 surface staining compared with the control group. The X axis shows fluorescence intensity and the Y axis shows cell number. The green curve represents the negative control and the red area represents the LAMP1 surface staining group.

### Transfections with siRNA

Cells were transfected in 1 ml Optimen (Gibco) with 50 nM siRNA (Invitrogen) using 5 μl Lipofectamine 2000 (Invitrogen). The siRNA sequences were from Santa Cruz Co. Transfections were interrupted after 6 h by adding 1 ml medium supplemented with 10% fetal bovine serum. The pre-microRNA sequences that targeted the human Rab27a gene plasmid were kindly provided by Dr R D Burgoyne from the University of Liverpool. We transfected the recombinant plasmids into glioma cells as described above, and this suppressed their Rab27a expression as determined by Western blotting.

**Figure 8:**
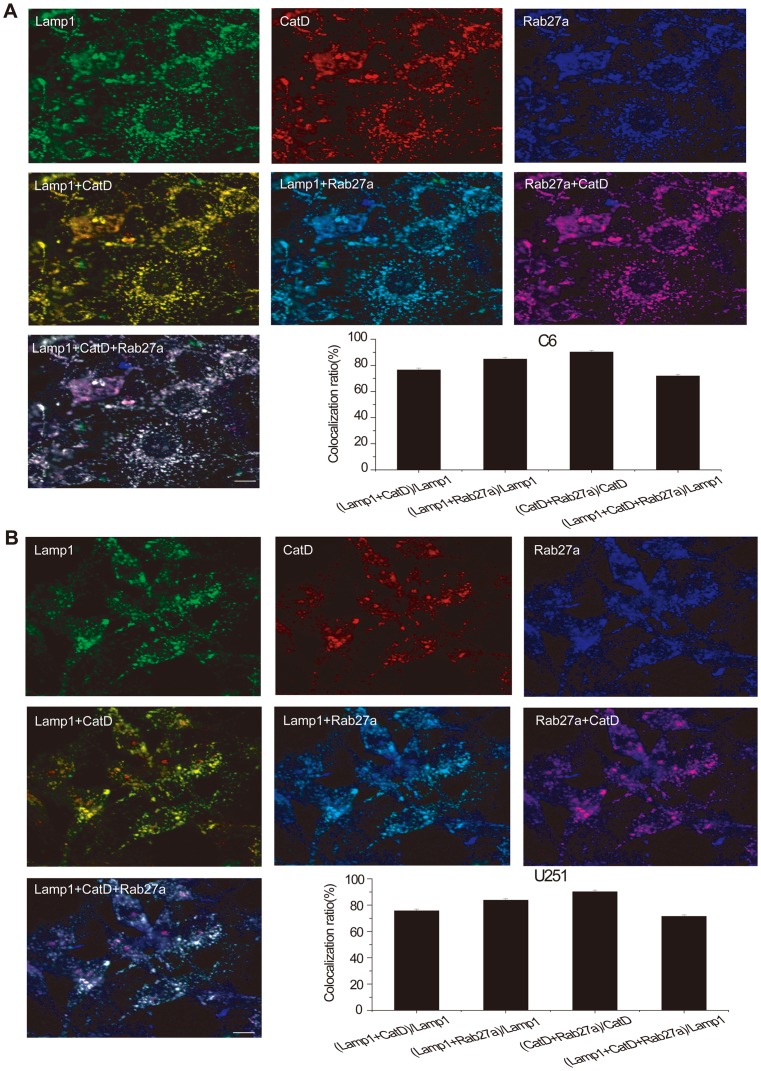
Confocal images showing lysosomes in the permeabilized C6 (A) and the permeabilized U251 (B) glioma cell lines containing cathepsin D and colocalized with Rab27a. C6 and U251 cells were immunostained with LAMP1 (green), cathepsin D (red), and Rab27a (blue). Bar charts show the percentage of colocalized puncta (labeled by both types of fluorescent dye). The number associated with each column refers to the number of image fields examined for each condition.

### Reverse transcription (RT)-PCR

The RNA from C6 and rat astrocyte was extracted using TRIzol® Reagent (Invitrogen). The quality and concentration of the RNA were determined by measuring the absorbance at 260 and 280 nm, and RNA integrity was confirmed by 1% agarose gel electrophoresis. First strand cDNA was synthesized using SuperScript III First-Strand Synthesis System for RT-PCR (Invitrogen). For control reaction, substitute 1 µl of DEPC-treated water for 1 µl of SuperScript III Reverse Transcriptase. Rab27a Primer sequences are: sense strand: 5′to3′GCATTGATTTCAGGGAAAAGAGAG; and antisense strand: 5′to3′TTGTCCACACACCGCTCCATCCGC.

The PCRs used 50 µl PCR media containing 5 µl of 10× PCR buffer, 3.0 µl 25 mM MgCl2, 1.0 µl 10 mM dNTP mix, 1.0 µl sense and antisense strand primers, 5 µl cDNA, 0.5 µl rTag, and 33.5 µl ddH2O, which underwent 94°C for 3 minutes, followed by 35 cycles of PCR amplifications under the following conditions: 94°C for 40 s, 62°C for 40 s, and 72°C for 60 s. The PCRs using the astrocyte or C6 RNA which had not been subjected to the reverse transcription were conducted to control for the potential genomic DNA contamination. PCR products were analyzed by 1% agarose gel electrophoresis and visualized by florescent DNA gel staining.

### Western blot

C6 glioma cell lines were cultured in 6-well plates until they reached 70–80% confluence. Cells were scraped and homogenized with lysis buffer containing 150 mM NaCl, 100 mM Tris-HCl, pH 8.0, 1 mM EDTA, 0.5% Triton X-100, and a protease inhibitor cocktail (Roche Diagnostics GmbH, Mannheim, Germany). Cells transfected with siRNA were lysed in the same way 2–3 days after transfection. The amount of soluble protein was determined using a modified Lowry assay (Bio-Rad, Richmond, CA). Twenty micrograms of total protein was loaded into each lane. Proteins were electrophoretically resolved by 9% sodium dodecyl sulfate polyacrylamide gel electrophoresis (SDS-PAGE) and transferred to polyvinylidene difluoride membranes (Immobilon PVDF; Millipore, Bedford, MA) by Western blotting. After blocking with 5% dry milk for 1 h at room temperature, PVDF membranes were incubated overnight with a 1∶200 dilution of Rab27A rabbit antibody (Santa Cruz). Subsequently, membranes were washed three times with PBS, and incubated for 1 h with 1∶2000 sheep anti-rabbit horseradish peroxidase-conjugated second step antibody (Amersham, Arlington Heights, IL). This was followed by five washes. Protein bands were visualized by an enhanced chemiluminescence Western blotting detection system (Amersham) and recorded on X-ray film (Kodak Biomax; Kodak, Rochester, NY). Molecular weights of the protein bands were assigned using the prestained protein ladder (BenchMark).

### Cathepsin D activity assay

The cathepsin D activity assay kit (Biovision) is fluorescence-based and uses the preferred cathepsin D substrate sequence labeled with MCA. Culture cell medium containing cathepsin D cleaved the synthetic substrate to release fluorescence, which was quantified using a fluorescence plate reader at Ex/Em 328/460 nm.

### Immunocytochemistry

Cells growing on a coverslip were fixed in 4% paraformaldehyde (PFA) in phosphate-buffered saline (PBS, pH 7.4) at room temperature (RT) for 10 min, and permeabilized by methyl alcohol at –20°C for 10 min before being blocked by 10% BSA for 1 h at RT. Cells were then stained with the following antibodies overnight at 4°C: mouse anti-LAMP1 (Assay Designs, VAM-EN001, 1∶200), goat anti- cathepsin D (R&D, AF1029,1∶200), rabbit anti-RAB27a (SantaCruz, SC-22756,1∶200). After washing to remove excess primary antibodies, the cultures were incubated for 1 h at RT with fluorescence-conjugated secondary antibodies: goat anti-mouse Alexa Fluor 488 (Invitrogen, 1∶1000), donkey anti-goat cy3 and goat anti-rabbit cy5 (Jackson Immuno research, 1∶1000). For the surface staining, cells were directly incubated with mouse anti-LAMP1 antibody (Assay Designs, VAM-EN001, 1∶200) overnight at 4°C without permeabilized. After staining with goat anti-mouse Alexa Fluor 488 secondary antibodies (invitrogen, 1∶1000), cells were permeabilized and total LAMP1 was stained by using rabbit anti-LAMP1 antibody (Abcam, ab24170, 1∶200) and goat-anti rabbit cy3 secondary antibodies (Jackson Immuno Research, 1∶1000). Images were captured on a confocal microscope (Olympus Fluoview 500-IX71). Scans from each channel were collected in multi-track mode to avoid cross-talk between channels. Data analysis was performed with Image-Pro Plus (MediaCybernetics, Bethesda, MD, USA).

### FACS

Monolayer cells were trypsinized using 0.25% trypsin-EDTA (Gibco) and then neutralized and resuspended with 10% FBS containing MEM (Gibco). After washing with cold PBS, cells were equally divided into two groups for each sample. One group was incubated with 50 μl mouse anti-LAMP1 antibody (Assay Designs, VAM-EN001, 1∶50) at 4°C for 1 h, and the other was incubated with the same amount of PBS. Cells were washed, fixed in 1% PFA and stained with goat anti-mouse Alexa Fluor 488 secondary antibody. Flow cytometry on at least 10,000 cells per sample was performed with a FACS (FC500MCL, Beckman Coulter) and data were analyzed by using CXP software (Beckman Coulter). Surface LAMP1 level was determined by the proportion of fluorescence positive cells compared to negative control.

### Calculations and statistics

Statistical analyses used ANOVA followed by Duncan's multiple-range test with p<0.05 as the level for significance.

## Results

### GPN and vacuolin-1 each decreased migration and invasion of C6 glioma cells

GPN is a lysosome-disrupting enzyme substrate which has been used to distinguish between lysosomal and prelysosomal compartments along the endocytic pathway. GPN causes rapid osmotic disruption of lysosomes due to intra-lysosomal accumulation of GPN cleavage products. Vacuolins induce the rapid formation of large, swollen structures derived from endosomes and lysosomes by homotypic fusion. Vacuolin-1, the most potent compound, blocks the Ca2+-dependent exocytosis of lysosomes. The images from the transwell experiments showed that the number of migrating cells decreased markedly in the presence of GPN or vacuolin-1 (Fig. 1A and D). The glioma cell invasion rate was inhibited 43% by 25 µM GPN and 33% by 1 µM vacuolin-1. These experiments were repeated with the C6 glioma cell line using either GPN concentrations ranging from 25 to 100 µM or vacuolin-1 concentrations from 1 to 4 µM (Fig. 1C and F). Both drugs showed dose-dependent inhibition of invasion. These doses were not evidently toxic to C6 cells (Fig. 1B and E).

### GPN and vacuolin-1 each decreased scratch-induced migration of C6 glioma cells

The movement of cells into the scarred region decreased the surface area of the scar after the 6-h incubation period (Fig. 2A). The overall change in the surface area of the scar for control, untreated samples was set to 100%, and the change in the surface area for samples treated with 100 µM GPN was 85.48±0.88%, a decrease of ∼15% (p<0.01), while 0.5 µM vacuolin-1 treatment resulted in a decrease of ∼12% (p<0.01) (Fig. 2B). The selective lysosome lysing drug GPN and the lysosome exocytosis inhibitor vacuolin-1 evidently inhibited migration and invasion in transwell experiments (Fig. 1) and scratch experiments (Fig. 2). The results suggest that inhibition of glioma cell lysosome exocytosis inhibits glioma migration and invasion.

### Reduced Rab27A expression attenuated the invasive potential of glioma cells *in vitro*

To avoid the possible problem of non-specific effects of the drugs, we constructed RNAi vectors targeting the Rab27a gene. Rab27a acts through organelle-specific effector proteins, such as granuphilin in pancreatic beta cells and melanophilin in melanocytes. The Rab27a and effector complex then interacts with proteins that are essential for membrane transport and fusion [9]. Both Rab27a shRNA and Rab27a siRNA decrease C6 migration 6 h after scratching (Fig. 3). Both Rab27a shRNA and Rab27a siRNA inhibited migration and invasion of C6 glioma cells in transwell experiments with (Fig. 4A and B) or without ECM gel (Fig. 4C and D). Reduced Rab27A expression resulted in a significant decrease in the invasive potential of C6 cells, both in the scratch and in the transwell assays.

To further confirm the results, we checked Rab27a-shRNA-GFP transfected cells in the scratch assay, and found that they migrated more slowly than the scrambled plasmid control group. The values at all three time points (6, 12, and 24 h) showed evident statistical differences (Fig. 3E). We repeated the same experiments in the U251 human glioma cell, and found similar results. The values at 6, 12, and 24 h also showed evident statistical differences (Fig. 3F). The data further confirmed that RNAi-Rab27A inhibit glioma cell migrtion in the *in vitro* assay.

RT-PCR (Fig. 5A) and Western blot (Fig. 5B) showed that Rab27a normally exists in C6 glioma cells. To confirm our PCR result, we sequenced the PCR product. The BLAST sequence showed that the identity with Rab27a was 100%. We demonstrated that the RNAi effectively reduced the endogenous Rab27a expression in these cells as detected by Western blotting (Fig. 5B). Approximately 50–60% reductions in Rab27A protein 48 to 72 h after transfection were found in RNAi-targeted C6 cells compared with non-silenced control.

### Reduced Rab27a expression decreased release of cathepsin D from C6 glioma cells. Inhibition of cathepsin D inhibited glioma cell migration

To further investigate the role of Rab27a in the inhibition of lysosome exocytosis, we determined the effect of its downregulation on cathepsin D release. With Rab27a siRNA or shRNA treatment, the release of cathepsin D was decreased compared to control, which meant that the lysosome enzyme exocytosis was inhibited by Rab27A downregulation (Fig. 5C).

In the scratch assay, when we inhibited cathepsin D activity by a chemical inhibitor pepstatin A, an inhibitor of aspartyl proteases to which cathepsin D belongs, both C6 and U251 glioma cell migration was inhibited. The values at 6 and 12 h showed evident statistical differences in both cell lines (Fig. 6).

### More lysosomes were located on the cell surface of glioma cells than on astrocytes. GPN decreased lysosome appearance on the cell surface. Cathepsin D was colocalized with Rab27a in the glioma cell lysosome

We compared the expression of the lysosome marker LAMP1 between C6 glioma cells and astrocytes by surface staining, and found that LAMP1 was expressed more on the surface of glioma cells than on astrocytes (Fig. 7A). To further support the surface staining result, we carried out a standard FACS assay for LAMP1 on the surface of C6 and U251 cells and astrocytes, and found both glioma cells had more lysosomes on the cell surface than astrocytes, according to the proporation of surface LAMP1 positive cells (Fig. 7B). After GPN treatment, the surface LAMP1 staining was decreased in C6 cells (Fig. 7B). These results suggested that the effect of GPN on glioma migration and invasion was links to lysosome exocytosis from glioma cells.

We also found large amounts of cathepsin D within glioma cell lysosomes in the permeabilized cells. It was interesting that Rab27a colocalized with cathepsin D and LAMP1 in both permeabilized C6 and U251 cells (Fig. 8). The result suggested that the action of Rab27a on lysosome cathepsin D exocytosis involved processes within the cytoplasm.

## Discussion

Lysosomes participate in the regulation and function of matrix metalloproteinases, serine proteases, and cathepsins, which are sequestered in lysosomal vesicles [10]. A recent study demonstrated that lysosomes are responsible for exocytosis in nonsecretory cells, such as astrocytes [12]. We found more lysosomes on the glioma cell surface than in astrocytes. We hypothesized that inhibiting lysosome exocytosis would reduce cathepsin D release and lead to reduced cancer invasion [13], [14]. Our results from the scratch motility assay indicated that GPN or vacuolin-1 modulated glioma cell movement in the two-dimensional plane. In the brain, such movement is not likely. Instead, glioma cells must be able to migrate and invade through the three-dimensional spatial constraints of the surrounding brain tissue. To replicate this type of environment more closely, we used a transwell migration assay and found that either GPN or vacuolin-1 inhibited glioma cell invasion in this three-dimensional model.

GPN, a cathepsin-C substrate, specifically induces extensive rupture of lysosomes without evident cell toxicity at low dosages. GPN has no effect on either autophagosomes or mitochondria. The ability of GPN to selectively destroy lysosomes makes it a useful aid in testing lysosome exocytosis effects [12]. Here, we treated the C6 glioma cell line with GPN at doses of 25–100 µM, and the LDH assay showed no toxicity. We found that GPN inhibited glioma cell migration and invasion, GPN decreased the number of lysosome on glioma cell surface, which means that it selectively disrupted lysosomes, inhibited lysosome exocytosis and inhibited glioma invasion.

Vacuolin-1 blocks the Ca^2+^-dependent exocytosis of lysosomes, without affecting the process of resealing [15]. In contrast, other cell structures and membrane trafficking functions, including exocytosis of enlargeosomes, are unaffected [16]. Here, we treated the C6 glioma cell line with vacuolin-1 (0.5–4 µM), and the LDH assay showed no toxicity. We found that vacuolin-1 inhibited glioma cell migration and invasion, which confirmed again that selective inhibition of lysosome exocytosis inhibited glioma invasion.

One paper reported that vacuolin-1, despite altering lysosome morphology, does not inhibit the exocytosis of lysosomes induced by exposure to a Ca^2+^ ionophore, or by plasma membrane wounding [17]. To resolve this conflict, and avoid possible side-effects, we used RNAi to downregulate Rab27a, a key protein in lysosome exocytosis [8], [9], [18], [19], and determine its effect on glioma invasion. It has recently been shown that the monomeric GTPase Rab27 subfamily regulates the exocytosis of cell-specific storage organelles [18]. Genetic alterations of Rab27a cause Griscelli syndrome in humans that manifests as pigmentary dilution of the skin and the hair and variable immunodeficiency due to defects in the transport of melanosomes in melanocytes and lytic granules in cytotoxic T-lymphocytes [20]. Recently, one report showed that Rab27a affects the invasive and metastatic potential of breast cancer cells by modulating the secretion of IGF-II, which regulates the expression of p16, VEGF, uPA, cathepsin D, cyclin D1, and MMP-9 [21]. In the present study, we found that Rab27a colocolized with cathepsin D in glioma cell lysosomes. We down-regulated the expression of Rab27a protein and explored its roles in the invasion and migration in glioma cells. Our results indicated that Rab27a played a positive regulatory role in invasion and metastasis and RNAi-Rab27a decreased the extracellular release of cathepsin D from these cells. When cathepsin D was inhibited by a chemical inhibitor, glioma cell migration was inhibited in the scratch assay. Since cathepsin D is a normal and major component of lysosomes, frequently used as a marker [22], these results confirm that the mechanism by which RNAi-Rab27a inhibits glioma invasion is by inhibiting lysosomal exocytosis of cathepsin D or the extracellular release of other lysosomal enzymes.

In conclusion, our results demonstrate that the inhibition of lysosome exocytosis by chemicals or RNAi inhibits glioma cell migration and invasion. Such effects of the inhibition of lysosome exocytosis have not been demonstrated previously in human brain malignancies. A better understanding of the mechanisms involved in this effect would not only contribute to our knowledge of the events that lead to the spread of human malignant glioma but would also apply to other cancers which migrate within different organs. These findings may lead to a new therapeutic strategy against glioma. However, further studies are necessary to understand the underlying mechanisms that mediate exocytosis from lysosomes during metastasis.
